# Role of oxalic acid in fungal and bacterial metabolism and its biotechnological potential

**DOI:** 10.1007/s11274-024-03973-5

**Published:** 2024-04-25

**Authors:** Marcin Grąz

**Affiliations:** Department of Biochemistry and Biotechnology, Institute of Biological Sciences, Maria Curie-Skłodowska University, Akademicka 19, 20-033 Lublin, Poland

**Keywords:** Oxalic acid, Oxalates, Oxalate decarboxylase, Oxalate oxidase, Oxalate-carbonate pathway, Fungi

## Abstract

Oxalic acid and oxalates are secondary metabolites secreted to the surrounding environment by fungi, bacteria, and plants. Oxalates are linked to a variety of processes in soil, e.g. nutrient availability, weathering of minerals, or precipitation of metal oxalates. Oxalates are also mentioned among low-molecular weight compounds involved indirectly in the degradation of the lignocellulose complex by fungi, which are considered to be the most effective degraders of wood. The active regulation of the oxalic acid concentration is linked with enzymatic activities; hence, the biochemistry of microbial biosynthesis and degradation of oxalic acid has also been presented. The potential of microorganisms for oxalotrophy and the ability of microbial enzymes to degrade oxalates are important factors that can be used in the prevention of kidney stone, as a diagnostic tool for determination of oxalic acid content, as an antifungal factor against plant pathogenic fungi, or even in efforts to improve the quality of edible plants. The potential role of fungi and their interaction with bacteria in the oxalate-carbonate pathway are regarded as an effective way for the transfer of atmospheric carbon dioxide into calcium carbonate as a carbon reservoir.

## Introduction

Oxalic acid (COOH)_2_, with the chemical systematic name ethanedioic acid, is a widespread organic acid in the biosphere. It is found in plants, animals, bacteria, and fungi as well as in soil and minerals (Kumar et al. 2019; Hervé et al. 2016; Gadd et al. 2014). Oxalic acid is one of the strongest carboxylic acids (Morrison and Boyd 1992). It occurs in nature both as insoluble salts formed with divalent metals such as calcium or copper and in a soluble form mainly as salts of sodium and potassium or as a free acid. Oxalic acid also has good chelating properties with divalent ions like manganese or ferric ions. Oxalic acid and its salts are low molecular weight compounds involved indirectly in many processes, e.g. pollutant bioremediation, wood degradation, biodeterioration processes, and rock weathering processes (Janusz et al. 2017; Gadd 2007; Goodell et al. 1997). Due to their chelating ability, oxalates are considered as good factors in the detoxification of heavy metals, including aluminium, lead, copper, and cadmium ions (Vlasov et al. 2023; Grąz et al. 2009, 2011). They participate in the biogeochemical cycles of certain nutrients and influence their bioavailability (Gadd 2007). The high affinity of oxalic acid for calcium ions should be emphasised. The formation of calcium oxalates in the human organism is considered as a serious problem leading to renal failure (Demoulin et al. 2022). Calcium oxalate crystals are also observed in fungi and plants (Gadd et al. 2014; Gadd 2007). Oxalates participate in fungal phytopathogenesis (McCaghey et al. 2019). The interactions between calcium and carbon in terrestrial ecosystems are linked, among others, in the oxalate-carbonate pathway (OCP), which has received considerable interest due to its potential role as a sink for carbon in the mineral form in soil (Syed et al. 2020). It seems increasingly evident that oxalic acid may play an important role in interactions between microorganisms living in the same ecological niche and in processes involved in biomineralisation, metal mobilisation, and nutrient availability.

## Role of oxalates in nutrient availability, biomineralisation, weathering of minerals, and metal precipitation

**Fig. 1:**
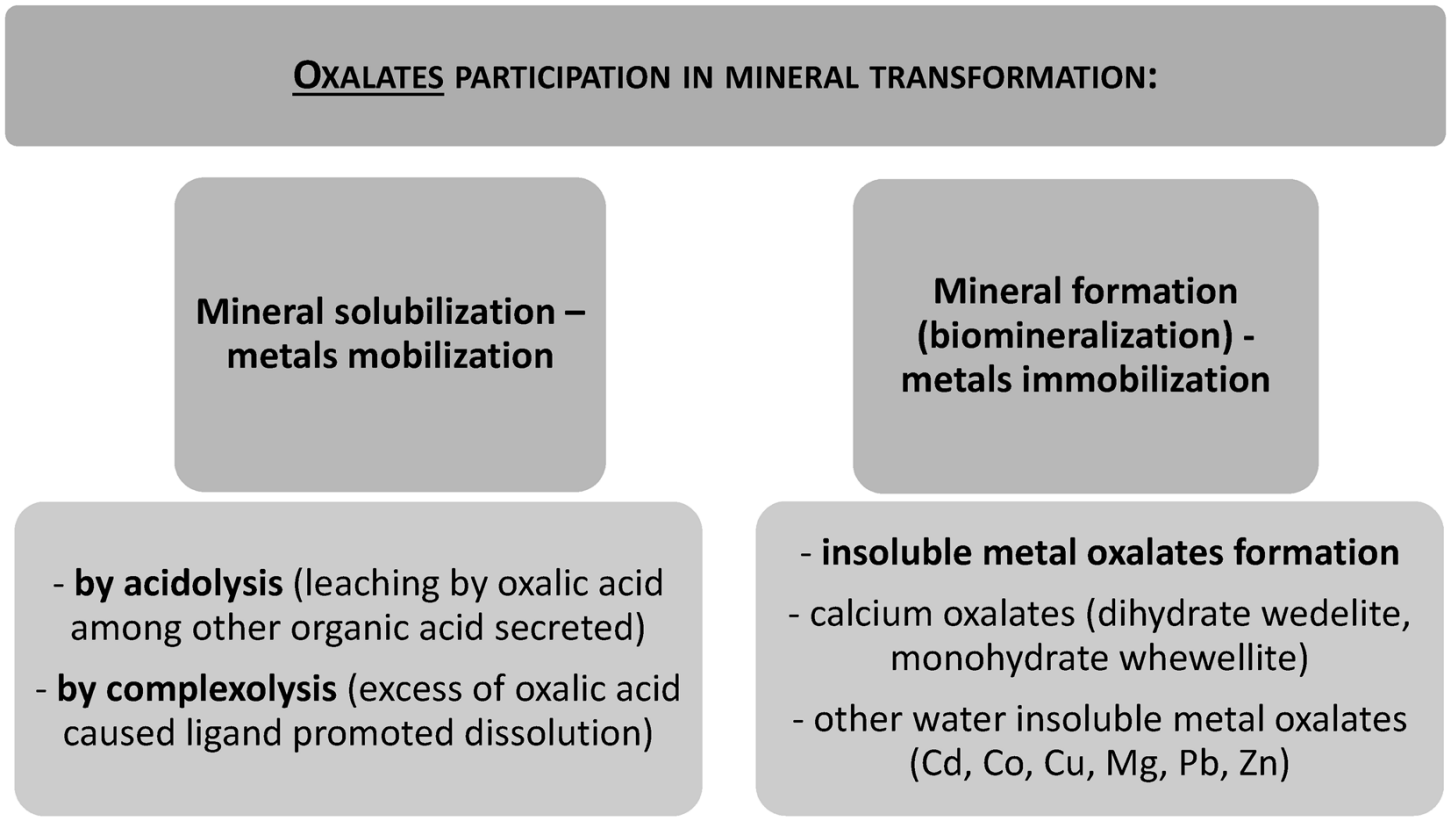
Roles of oxalates in mobilisation and immobilisation of metals

Fungi have a significant impact on ecosystem functioning by participating in biogeochemical cycles related to the availability of especially carbon, nitrogen, and phosphorus (Yin et al. 2022; Liu et al. 2021; Gadd 2007). Fungi can be considered as the largest group of microorganisms on the Earth’s surface in terms of biomass. This, together with their branching growth mode, makes fungi an efficient element in the mobilisation and immobilisation of metals in the environment (Bohu et al. 2022; Gadd 2007). The role of fungi in bioweathering of minerals includes the direct biomechanistic action of the mycelium itself and the indirect action associated with fungal metabolites. The indirect biochemical mode of action is related to fungal exudates and involves such compounds as organic acids, oxidising or reducing agents, or ligands like siderophores (Muksy et al. 2023). Oxalic acid is often detected in the fungal secretome, and many of the processes involved in biomineralisation and bioweathering are related to this simple organic acid (Sindhu et al. 2022). The biotic weathering involving the action of fungi may be the source of essential elements, such as K, P, and Ca, and many trace elements, including transition metals (Bohu et al. 2022; Sui et al. 2021; Hervé et al. 2021). Oxalic acid has potential as a metal chelator. It can mediate mineral dissolution through the mechanism of acidolysis and complexation. In the context of new mineral formation called biomineralisation, oxalates can form a cooper oxalate precipitant named moolooite as well as insoluble calcium salts in the form of dihydrate wedelite and monohydrate whewellite. These provide a reservoir of calcium in the environment and affect the availability of phosphate (Gadd et al. 2014; Gadd 2007). The oxalate-mediated formation of salts with divalent metals also underpins the mechanism of heavy metal tolerance by fungi. Insoluble oxalate salts include salts with divalent metals, such as cadmium, cobalt, copper, magnesium, lead, strontium, and zinc (Vlasov et al. 2023; Frank-Kamenetskaya et al. 2021; Jarosz-Wilkołazka and Graz 2006). The aerobic mode of growth of fungal mycelium can also be a source of carbon dioxide, which can lead to the formation of carbonic acid in the soil. Consequently, the dissolution of minerals present in the soil may be promoted due to changes in the pH value caused by carbonic acid and by extraction of divalent metals precipitated as oxalates (Kang et al. 2023; Gadd et al. 2014). The roles of oxalates in these processes are shortly summarised in Fig. 1. In this context, oxalate can be considered as an important fungal metabolite influencing some important biological processes referred to as geomycology, including but not limited to bioweathering and biomineralisation (Gadd et al. 2014; Gadd 2007).

## Oxalate as part of lignocellulolytic machinery

**Fig. 2:**
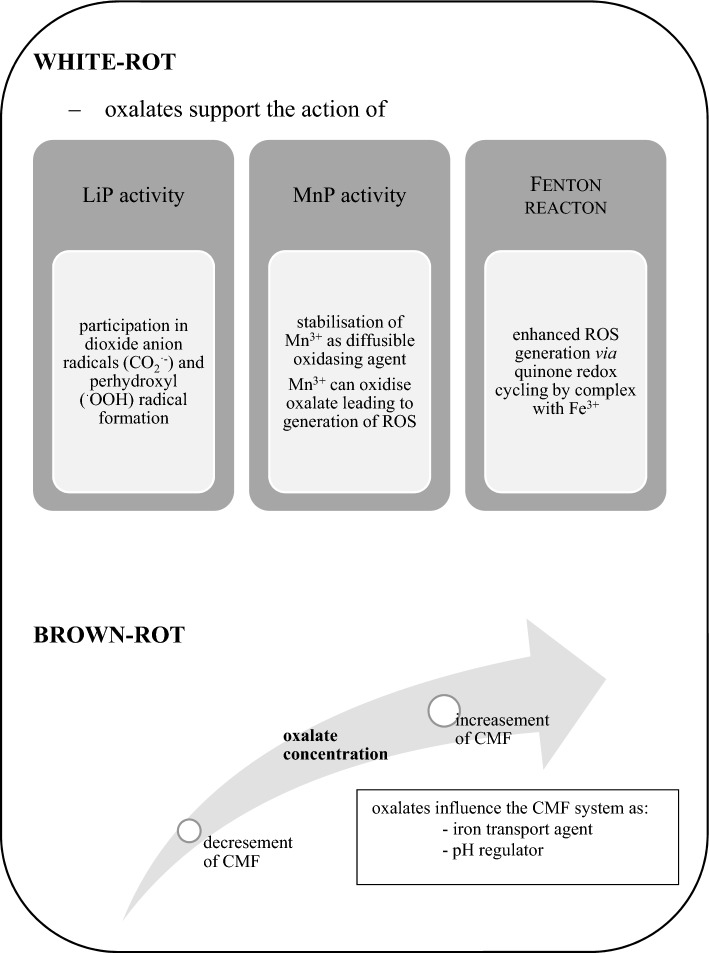
Participation of oxalates in the lignocellulolytic system of white and brown rot fungi. *LiP* lignin peroxidase, *MnP* manganese-dependent peroxidase, *ROS* reactive oxygen species, *CMF* chelator-mediated Fenton system

Fungi, especially those causing white rot of wood, are more efficient in the breakdown of the lignocellulose complex than bacteria. Bacterial delignification is thought to be more limited than the fungal process (Grgas et al. 2023; Asina et al. 2016). The main parts of the fungal lignin degradation system are enzymes, which can be divided into two groups: lignin-modifying enzymes and lignin-degrading auxiliary enzymes (Janusz et al. 2017). The most important of the lignin-modifying enzymes are laccases (Lac, EC 1.10.3.2) and peroxidases, e.g. lignin peroxidase (LiP, EC 1.11.1.14), manganese-dependent peroxidase (MnP, EC 1.11.1.13), versatile peroxidase (VP, EC 1.11.1.16), and dye-decolourising peroxidase (DyP, EC 1.11.1.19) (Janusz et al. 2013, 2020). The many lignin-degrading auxiliary enzymes are predominated by those providing hydrogen peroxide needed for peroxidases, i.e. aryl alcohol oxidase (EC 1.1.3.7), pyranose 2-oxidase (EC 1.1.3.10), and glyoxal oxidase (EC 1.2.3.5) as well as such dehydrogenases as glucose dehydrogenase (EC 1.1.99.10) and cellobiose dehydrogenase (EC 1.1.99.18) (Sulej et al. 2021; Chan et al. 2020). Low molecular weight compounds are considered as a very important component of the fungal machinery involved in the degradation of the lignocellulose complex. At the early stage of this process, they act as diffusible agents reacting directly with the lignocellulose complex, which makes them accessible to the action of enzymes (Janusz et al. 2017). The low molecular weight compounds include reactive oxygen species (ROS), extracellular aromatic compounds, transition metals, and organic acids (Janusz et al. 2017; Arantes et al. 2012). Most filamentous fungi are able to acidify their growth medium by secreting organic acids, e.g. gluconic, formic, oxalic, malic, succinic, citric, lactic, acetic, and glyoxylic acids (Narisetty et al. 2023; Liaud et al. 2014; Jarosz-Wilkołazka and Graz 2006; Mäkelä et al. 2002). Oxalic acid plays a role in the mechanism of both brown and white rot wood decay (Füchtner et al. 2023; Presley et al. 2018; Arantes et al. 2012). In white rot fungi, oxalate serves as a chelator of Mn^3+^ ions, which are formed in the catalytic cycle of MnP and serve as diffusible oxidising agents able to attack phenolic compounds contained in the lignin structure (Bilal et al. 2023; Kumar and Arora 2022). In brown rot fungi, the mechanism of the attack on the lignocellulose complex consists in selective removal of carbohydrates. This two-step action combines non-enzymatic activity prior to enzymatic hydrolysis. Non-enzymatic depolymerisation of wood cell wall polysaccharides involves low molecular weight oxidants generated in a chelator-mediated Fenton system (CMF). Oxalates play an important role in this process as iron transport agents, pH regulators, and physiological iron reductants (Zhu et al. 2016; Arantes and Goodell 2014). The concentration of oxalic acid is important in the control of CMF-based reaction. Oxalates enhance Fenton reaction in a low concentration but weaken this process in a higher concentration, which is related to pH gradient generation and dependent formation of iron mobilising Fe^3+^-oxalate complexes versus Fenton inhibiting Fe^3+^/Fe^2+^-(oxalate)_2,3_ complexes (Presley et al. 2018; Zhu et al. 2016). The oxalate concentration in fungal vicinity is then actively regulated via enzymatic action of mainly oxalate decarboxylase (Svedružic et al. 2005; Mäkelä et al. 2002). Recently, accumulation of oxalates also inside the cell wall of wood infected by brown-rot fungus *Rhodonia placenta* has been observed (Füchtner et al. 2023). Oxalate is not the only low molecular weight agent involved in the Fenton-based oxidation process in brown rot fungi. Fungal hydroquinones, for example 2,5-dimethoxyhydroquinone, can also be mentioned here as part of the ROS generating system in the redox cycling process (Presley et al. 2018; Krueger et al. 2016). White rot fungi also generate ROS during lignocellulose degradation, and the Fenton reaction takes part in lignin complex degradation synergistically with fungal oxidative enzymes (van der Made et al. 2023; Merino et al. 2020). In white rot fungi, the induction of generation of hydroxyl radicals via quinone redox cycling is enhanced in the presence of oxalate as a Fe^3+^-oxalate complex (Gomez-Toribio et al. 2009a, b). Oxalates also take part in the dioxide anion radicals (CO_2_^·−^) and perhydroxyl (^·^OOH) radical formation through the oxidation of oxalate in a LiP reaction mediated by veratryl alcohol. It can result in further Mn^3+^ formation. In the MnP catalytic cycle, Mn^3+^ ions can oxidise oxalates, leading to the generation of ROS, which cause further non-specific chemical oxidation reactions (Hammel and Cullen 2008; Popp et al. 1990). This and the aforementioned role of oxalates as chelators of Mn^3+^ ions generated in the MnP cycle makes oxalates an important factor in the mechanisms of the lignocellulose degradation system in white rot fungi (Fig. 2). It should be pointed out that the Fenton reaction coupled with enzymes of white rot fungi can be a powerful tool employed in many environmental applications concerning degradation of organic pollutants (Chen et al. 2023; van der Made et al. 2023).

An important aspect of the role of oxalic acid is its interaction with fungal extracellular matrix (ECM). The fungal ECM plays an important role in the biomass conversion efficiency. Soluble oxalate cross-links with β-glucan, which is the main component of fungal ECM, to form a hydrogel. The concentration of oxalic acid changes the physical properties of the β-glucan gel. Therefore, the secretion of oxalic acid allows fungi to control the diffusion of large proteins, including ligninocellulotic enzymes (Peter-Gonzalez et al. 2023).

## Microbial synthesis of oxalic acid and enzymes involved in this process

**Fig. 3:**
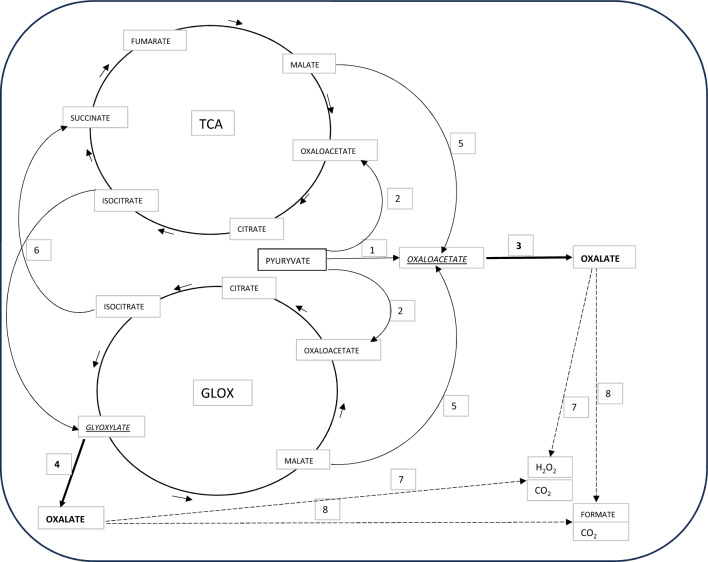
Fungal biochemical pathways of oxalic acid biosynthesis and degradation. 1—pyruvate carboxylase (EC 6.4.1.1); 2—pyruvate dehydrogenase (EC 1.2.5.1); 3—oxaloacetase (EC 3.7.1.1); 4—glyoxylate dehydrogenase (EC 1.2.1.17); 5—malate dehydrogenase (EC 1.1.1.37); 6—isocitrate lyase (EC 4.1.3.1); 7—oxalate oxidase (EC 1.2.3.4); 8—oxalate decarboxylase (EC 4.1.1.2)

Fungi secrete oxalic acid in large quantities into the culture medium (Hastrup et al. 2012; Plassard and Fransson 2009; Jarosz-Wilkołazka and Graz 2006). It can be secreted by fungi in the form of oxalate salts, because the pH values of cultures are often not altered despite the detection of oxalate ions in the medium (Hastrup et al. 2012; Grąz and Jarosz-Wilkołazka 2011). Oxaloacetic acid and glyoxylic acid are the direct precursors of oxalic acid (Fig. 3). The main enzymes involved in direct oxalic acid biosynthesis are therefore oxaloacetase (OXA, EC 3.7.1.1) and glyoxylate dehydrogenase (GLOXDH, EC 1.2.1.17). It is considered that the pathway of oxalic acid biosynthesis involving OXA is localised in the cytoplasm, and the activity of GLOXDH is peroxisomal (Zhuang et al. 2015; Sakai et al. 2006). In the GLOX cycle, GLOXDH directly oxidises glyoxylic acid to oxalic acid. In the cytoplasmic pathway, the direct precursor of oxalic acid, i.e. oxaloacetate, can be formed from pyruvate, which is carboxylated into oxaloacetate via the action of pyruvate carboxylase (PC, EC 6.4.1.1), or is derived from the tricarboxylic acid pathway (TCA) or the glyoxylate pathway (GLOX). In such a case, oxaloacetate is formed from malate in the reaction catalysed by malate dehydrogenase (MD, EC 1.1.1.37) (Fig. 3). In the context of oxalic acid biosynthesis, both TCA and GLOX cycles are coupled in fungal metabolism (Zhuang et al. 2015; Sakai et al. 2006; Munir et al. 2001). Generally, the GLOX cycle is metabolically activated during glucose starvation, but in *Fomitopsis palustris*, the GLOX cycle operates even in the presence of glucose, and a strong correlation was detected between glucose consumption and oxalate production in this fungus (Munir et al. 2001). An important enzyme which coupling metabolites in both the TCA and GLOX cycles is isocitrate lyase (ICL, EC 4.1.3.1), which cleaves the C–C bond of the isocitrate molecule forming succinate and glyoxylate capable of supplementing both cycles (Munir et al. 2001). The biosynthesis of oxalic acid, similar to other low molecular weight organic acids in fungi, may be affected by some factors, e.g. the available source of carbon or nitrogen, the pH value of the medium, the presence of divalent cations, the stage of cultivation, and the carbon/nitrogen (C/N) ratio (Plassard and Fransson 2009). In *Gleophyllum trabeum* cultures, the C/N ratio is a key factor in oxalate biosynthesis. In high nitrogen conditions, the cytosolic oxaloacetase pathway dominates, in contrast to low nitrogen conditions, where the peroxisomal glyoxylate dehydrogenase pathway is predominant (Zhuang et al. 2015). In the secretome of *Bjerkandera fumosa*, a higher concentration of oxalic acid was observed in the conditions without nitrogen limitation (Grąz and Jarosz-Wilkołazka 2011). In the brown rot fungus *Fomitopsis palustris*, the biosynthesis of oxalic acid and the activity of enzymes required for the glyoxylate cycle were greater at the vegetative growth stage of the fungus than at the fruiting stage (Yoon et al. 2002). In old mycelia of *Sclerotinia sclerotiorum*, both the oxaloacetase gene expression and accumulation of this protein were strongly repressed (Wang et al. 2016). The oxalic acid accumulation in cultures of *Sclerotinia sclerotiorum* D-E7 was suppressed in conditions below pH 4 (Culbertson et al. 2007). This is in agreement with the observation of *Abortiporus biennis* cultures, where oxalate oxidase activity correlated with a decreased oxalic acid concentration was observed only in conditions under pH 4 (Grąz et al. 2016). Regarding bacteria, there is a gap in the knowledge of oxalic acid biosynthesis. It was demonstrated in *Burkholderia glumae* that oxalic acid biosynthesis is encoded by an operon. Two transcripts *obcA* and *obcB* coexpressed and encoded on a single polycistron message were demonstrated (Nakata and He 2010).

## Oxalic acid degradation by fungi and bacteria

The enzymatic degradation of oxalic acid takes place via its decarboxylation or oxidation (Fig. 3). The reactions can be carried out using the oxalic acid molecule or its form activated by the CoA molecule. The degradation of oxalic acid via decarboxylation is typical for fungi and is catalysed by a lyase named oxalate decarboxylase (ODC, EC 4.1.1.2). The activity of ODC leads to the decarboxylation of oxalic acid to formic acid and carbon dioxide. The best-characterised ODC are those from basidiomycete fungi *Flammulina velutipes*, *Dichomitus squalens,* and *Sclerotinia sclerotiorum* (Liang et al. 2015a, b; Mäkelä et al. 2009; Chakraborty et al. 2002) as well as *Phanerochaete sanguinea*, *Trametes ochracea*, *Trametes versicolor*, *Bjerkandera fumosa*, *Agaricus bisporus*, *Gleophylum trabeum*, *Pleurotus ostreatus*, *Postia placenta*, *Schizophyllum commune*, and *Serpula lacrymans* (Grąz et al. 2011; Mäkelä et al. 2002, 2010). Fungi also employ the oxidative pathway of oxalic acid degradation catalysed by oxalate oxidase (OXO, EC 1.2.3.4). The activity of this oxidoreductase leads to the oxidation of oxalic acid to hydrogen peroxide and carbon dioxide. This oxidative pathway is mainly found in plants. In fungi, OXO has been found and characterised so far in *Ceriporiopsis subvermispora* and *Abortiporus biennis* (Grąz et al. 2016, 2020; Escutia et al. 2005; Aguilar et al. 1999). The activity of oxalate oxidase was also confirmed in *Schizophyllum commune*, *Trametes hirsuta*, *Gloeophyllum trabeum*, *Abortiporus biennis*, *Cerrena unicolor*, *Ceriosporopsis mediosetigera*, *Trametes sanguinea*, *Ceriporiopsis subvermispora*, and *Laetiporus sulphureus* (Grąz et al. 2023). Both oxalate decarboxylase and oxalate oxidase represent cupins, which are a superfamily of proteins with various enzymatic and non-enzymatic functions (Svedružic et al. 2005; Dunwell et al. 2004). The process of oxalate degradation in bacteria needs the activation of the oxalic acid molecule by CoA giving oxalyl-CoA as a substrate for thiamine pyrophosphate (TPP)-dependent oxalyl-Co decarboxylase (EC 4.1.1.8), yielding formyl-CoA and carbon dioxide (Svedružic et al. 2005). It was also found in *Moorella thermoacetica* that oxalic acid can be degraded through oxidation by TPP-dependent oxalate oxidoreductase (OOR) generating two CO_2_ molecules and two low-potential electrons (Gibson et al. 2016).

The evidence that oxalate production is coupled with energy production was provided for brown-rot fungus *Fomitopsis palustris* (Munir et al. 2001). In this fungus, during a process named oxalate fermentation, biochemical energy is acquired by oxidising glucose to oxalate. The proposed mechanism involves the TCA and GLOX cycles, which are both anaplerotic to each other, and is based on the oxidation of acetyl-CoA to yield oxalate. In the proposed system, malate dehydrogenase (MD, EC 1.1.1.37) is recognised as an enzyme which plays a major role in generating energy due to the NADH production in the reaction of oxidation of malate to oxaloacetate. It is important that another energy equivalent in the form of NADH can also be generated through the action of the enzyme formate dehydrogenase (FDH, EC 1.2.1.2) identified in white rot fungi, which oxidises oxalate to formate (Watanabe et al. 2005, 2008; Tishkov and Popov 2004). The acquisition of biochemical energy suggested by Munir et al. (2001) may be a general feature of basidiomycete fungi, but this thesis still needs to be confirmed. In energy acquisition by oxalotrophic bacteria, the transportation of oxalate across the membrane generates ATP. It is connected with the action of the membrane oxalate formate antiporter (oxlT) working together with the decarboxylation of CoA-activated oxalic acid. It develops the membrane potential, which is further used to drive H^+^-dependent ATPase (Hiremath and Viswanathan 2022).

## Potential biotechnological applications related to oxalate metabolism

Oxalate precipitates can affect some industry-related processes and become a problem, as in the case of e.g. precipitates in the Bayer process or in pulp bleaching (Cheng et al. 2023; Sousa et al. 2023). Industry-related processes will not be included in this review. In the following sections, we will focus more on the use of biological ways to prevent the accumulation of oxalate salts observed in kidney disease as well as its role in plant disease caused by *Sclerotinia sclerotiorum* and the role of oxalates in a process that can provide an efficient carbon dioxide reservoir, called the oxalate-carbonate pathway.

### Reduction of the risk of oxalate stone formation in kidney diseases—the use of oxalate-degrading and oxalotrophic bacteria as probiotics

In the human body, oxalates are present in blood plasma and urine as the end products of metabolism or originate from food rich in oxalic acid, e.g. rhubarb, spinach, tea, chocolate, and coffee. Disturbances in the regulation of both endogenous and exogenous oxalate concentrations can lead to primary or secondary hyperoxaluria. Modern increased dietary ingestion of oxalates linked with microbiota dysbiosis may lead to secondary hyperoxaluria, which is the main risk factor for calcium oxalate urolithiasis (Demoulin et al. 2022). Oxalates are adsorbed in the intestinal epithelium and, after precipitation with calcium in urine, can form oxalate kidney stones. Calcium oxalates are the most prevalent of kidney stones (Wigner et al. 2022). There are no enzymes found in humans for metabolising oxalates. It is believed that one of the effective ways of regulating their quantity in the body (apart from adsorption in the urinary tract or the formation of insoluble calcium oxalates) is elimination through the action of the microbiota found in the digestive tract (Karamad et al. 2022; Soliman et al. 2021; Giardina et al. 2014). Bacterial oxalotrophy has been well-recognised. Oxalotrophy may be defined as the ability of an organism to use oxalate or oxalic acid as carbon and electron sources. When considering oxalate as a carbon source in bacterial metabolism, two pathways for its assimilation are involved: the glycolate pathway and the serine pathway (Robertson and Meyers 2022; Sahin 2003). Oxalotrophic bacteria should be defined as a physiological rather than taxonomic group. Still, the best predictor of a strain's ability to degrade oxalate will therefore be the presence of genes for proteins involved in this process, especially enzymes mentioned previously in chapter 4 (Sahin 2003). The analysis carried out by Hervé et al. (2016) revealed that oxalate-degrading bacteria originated from terrestrial, aquatic, and clinical environments and were restricted to three phyla, namely Actinobacteria, Firmicutes, and Proteobacteria. The best-characterised bacterial species with the ability to use oxalates as an energy source is *Oxalobacter formigenes*. It is a Gram-negative, anaerobic, and nonpathogenic bacterium that converts oxalate into formate and carbon dioxide (Wigner et al. 2022). Because it is a strict anaerobe, effective manufacture of this strain poses many challenges (Wigner et al. 2022). The oxalic acid degradation pathway in *O. formigenes* is composed of the following proteins and their genes: oxalate/formate antiporter (oxlT), axalyl-CoA decarboxylase (oxdC), formyl-CoA transferase (frc) (Hiremath and Viswanathan 2022). *O. formigenes* demonstrates a unique ability among oxalotrophs to use oxalate from endogenous and exogenous sources. It has the ability to initiate a net intestinal oxalate secretion into the lumen from the bloodstream (Chamberlain et al. 2020). Increased attention is also paid to microorganisms that are able to degrade dietary oxalates for their potential application as probiotics. Among them, the genera *Lactobacillus* and *Bifidobacterium* are considered as a target for such studies. *Lactobacillus plantarum PBS067*, *Lactobacillus acidophilus LA-14*, *Bifidobacterium breve PBS077*, and *Bifidobacterium longum PBS078* were proposed as a tool for therapeutic treatment of hyperoxaluria as well as inflammatory events accompanying oxalate accumulation (Giardina et al. 2014). *Lactobacillus fermentum* NRAMJ5 and *Lactobacillus gastricus* NRAMJ2 were identified as good probiotic candidates for managing hyperoxaluria. These bacterial strains were able to produce exopolysaccharides, tolerated bile salt and acidity, and exerted an antagonistic effect against some pathogens, including bacteria and yeasts *S. cerevisiae*, *C. albicans*, and the filamentous fungus *A. niger* (Soliman et al. 2021). Beside *Lactobacillus* spp., bacteria classified in the genera *Clostridium* and *Enterococcus* were found to be capable of degrading oxalates (Miller et al. 2014). In many cases, this bacterial capacity has yet to be proven in in vivo studies. Recently, a synthetic biology approach has been developed by the construction of engineered bacteria (SYNB8802) able to consume oxalate in the gut and reduce urinary oxalate levels. The synthetic biotic SYNB8802 was constructed by cloning and expressing genes for oxalate/formate antiporter, oxalyl-CoA decarboxylase, and formyl-CoA transferase, i.e. well-known proteins involved in oxalate metabolism, but an additional gene for oxalyl-CoA synthetase was required to be co-expressed to reveal the ability to reduce the oxalate level by these engineered bacteria (Lubkowicz et al. 2022). Another approach to addressing oxalate deposit formation is to supplement patients with free oxalate-degrading enzymes, i.e. ODC and OXO. However, this approach requires effective ways to deliver the enzymes, and a promising way is to use both free enzymes and oxalotrophic microorganisms in treatments (Gupta and Kanwar 2020; Peck et al. 2016).

### Removal of oxalate as a virulence factor (e.g. *Sclerotinia sclerotiorum*)

The *Sclerotinia sclerotiorum* fungus belonging to Ascomycota is agriculture’s most devastating plant necrotrophic pathogen. For colonisation of host plant tissues, *S. sclerotiorum* requires, inter alia, oxalic acid, which is considered as a non-host specific toxin (Liang et al. 2015a, b). The role of oxalic acid in the pathogenicity of this necrotrophic fungus is well known but not fully understood (McCaghey et al. 2019). Nevertheless, it was demonstrated that mutants of the fungus lacking the ability to produce oxalic acid showed reduced pathogenicity (Rana et al. 2022; McCaghey et al. 2021; Liang et al. 2015a). The main roles of oxalic acid in the pathogenesis of *S. sclerotiorum* are the acidification of the environment in the middle lamellae, its function as a calcium ion chelating agent, and enhancement of the activities of cell wall depolymerising enzymes. Oxalic acid interacts with the ROS generating system and affects the host redox environment, thus lowering the response of the host defence system (Hossain et al. 2023; McCaghey et al. 2019). It has also been hypothesised that oxalic acid protects *S. sclerotiorum* mycelium from the toxic effects of excess calcium ions in the infection zone (Heller and Witt-Geiges 2013). The overexpression of oxalate oxidase or oxalate decarboxylase in plants increases their resistance against white mould caused by *S. sclerotiorum* (Verma and Kaur 2021; Kumar et al. 2016; Donaldson et al. 2001). In addition to the breakdown of oxalic acid, oxalate oxidase also generates hydrogen peroxide, which belongs to ROS and is a plant signalling molecule. In this way, it can influence the plant defence response, e.g. programmed cell death involved in the hypersensitive response of plant cells (Elena-Real et al. 2021).

### Oxalate-carbonate pathway as the metabolic interaction among fungi and bacteria—long term sink for atmospheric CO_2_

**Fig. 4:**
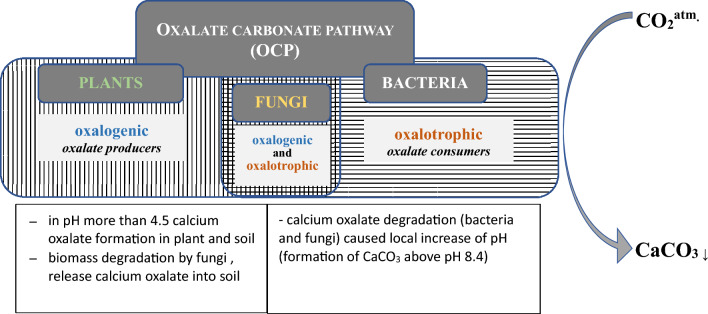
Oxalate carbonate pathway (OCP). Role of fungi as oxalate producers and degraders

The oxalate-carbonate pathway (OCP) is a biogeochemical process linking calcium oxalate oxidation and carbonate precipitation with generation of calcium carbonates (Fig. 4). The formation of carbonates is enabled by a local increase in the pH value in soil caused by the oxalotrophic activity of microorganisms. Since calcium carbonates are more stable (10^2^ – 10^6^ years) than biomass, the OCP can serve as an efficient carbon dioxide reservoir. For this reason, the OCP takes part in the global carbon cycle and is a potential long-term sink for atmospheric carbon dioxide (Syed et al. 2020). The OCP has been found mainly in tropical ecosystems so far (Hervé et al. 2016; Rowley et al. 2017). According to the current paradigm, the OCP involves oxalogenic and oxalotrophic organisms. Plants and fungi are classified as oxalogenic organisms, whereas bacteria are oxalate consumers. The taxonomy of bacteria and plants is known, but the taxonomy of fungi is rather poorly clarified. It is now postulated that fungi also play a role as an oxalotrophic part in the OCP pathway, and not just as oxalate producers (Hervé et al. 2016). The elucidation of the interactions between bacteria and fungi in the soil seems to be essential to fully understand environmental soil processes. A better understanding of the fungal species involved as well as their functions in the OCP pathway, together with the existing knowledge of the bacteria involved, will allow designing microscale experiments to better understand the mechanisms and regulation of the OCP process (Martin et al. 2012).

## Conclusion

Oxalic acid can be considered as an important component of the fungal secretome. The review presented here aimed to demonstrate the importance of oxalic acid and its salts in environmental processes involving both physicochemical and microbial processes. The study of the biological pathways of oxalate regulation in the microbial community remains still an important aspect. The study of oxalotrophic organisms or enzymes involved in oxalate synthesis and decomposition has potential applications in the diagnostic approach and therapy of kidney stone-related diseases. Undoubtedly, a new aspect concerning oxalic acid that needs to be better understood is its involvement in the OCP cycle, which is important given its role in the carbon cycle in nature.

## Data Availability

No datasets were generated or analysed during the current study.
